# Antifungal Efficacy of Essential Oils and Nanoformulations Against Fusarium Wilt of Tomato: Systematic Review (2000–2025)

**DOI:** 10.3390/plants15081268

**Published:** 2026-04-21

**Authors:** Salam Y. Abuzaitoun, Mazen N. Salman, Yamen Y. Hamdan, Rana M. Jamous, Mohammed S. Ali-Shtayeh

**Affiliations:** 1 Biodiversity and Environmental Research Center, Nablus P.O. Box 696, Palestine; rana.jamous@berc.ps (R.M.J.); mohd.saleem.shtayeh@berc.ps (M.S.A.-S.); 2 Department of Agricultural Biotechnology, Palestine Technical University, Tulkarm P.O. Box 7, Palestine; m.salman@ptuk.edu.ps (M.N.S.); y.hamdan@ptuk.edu.ps (Y.Y.H.)

**Keywords:** Fusarium wilt, *Fusarium oxysporum* f. sp. *lycopersici*, tomato, essential oils, nanoemulsions, nano-biopesticides, defense priming, sustainable plant disease management

## Abstract

*Fusarium oxysporum* f. sp. *lycopersici* (FOL) is one of the most destructive soil-borne pathogens affecting tomato production worldwide, causing substantial yield losses and persisting in soil for extended periods. The increasing regulatory restrictions on chemical fungicides and the emergence of resistant pathogen strains have intensified the search for sustainable and environmentally friendly alternatives. This systematic review synthesizes studies published between 2000 and 2025 that evaluated the antifungal efficacy of essential oils (EOs), their bioactive constituents, and EO-based nanoformulations against FOL in tomato. A total of 40 studies were included, following the PRISMA 2020 guidelines, encompassing in vitro, greenhouse, and limited field evaluations. Many EOs rich in phenolic compounds and oxygenated monoterpenes, such as thymol, carvacrol, eugenol, citral, and menthol, consistently inhibited FOL growth and spore germination, with reported mycelial growth inhibition ranging from 60 to 100% and minimum inhibitory concentrations (MICs) between 0.05 and 1.5 µL ml^−1^. However, the use of EOs is often limited because they evaporate quickly, do not mix well with water, can harm plants, and do not persist under field conditions. Nano-delivery systems, including nanoemulsions, polymeric nanoparticles, chitosan-based carriers, and lipid-based nanostructures, have been shown to enhance the stability, bioavailability, and antifungal efficacy of EOs. This has led to improved disease management and reduced pesticide application rates. In addition, several EO-based treatments have been reported to activate plant defense responses, including the induction of defense-related genes, antioxidant enzymes, and epigenetic modifications. Overall, EO-based nanoformulations show promise as next-generation biopesticides for the sustainable management of tomato Fusarium wilt. Nevertheless, large-scale field validation, standardized formulation protocols, and regulatory assessments are required before these technologies can be widely implemented in agriculture.

## 1. Introduction

Tomato (*Solanum lycopersicum*) is one of the most economically important vegetable crops worldwide. Global tomato production exceeds 180 million metric tons annually, making tomatoes one of the most widely cultivated vegetable crops [1]. Tomato cultivation is severely threatened by *Fusarium oxysporum* f. sp. *lycopersici* (FOL), the causal agent of Fusarium wilt disease. Fusarium wilt causes substantial economic losses, typically reducing yields by 30–40%, with losses exceeding 80% under favorable disease conditions [2]. Economic losses from Fusarium wilt range from 25 to 55% across various localities in India [3].

FOL infects tomato roots and attacks the xylem, causing vascular wilt symptoms, including yellowing, wilting, and frequent plant death [4]. The pathogen persists in the soil for many years through resilient chlamydospores, making eradication difficult once a field becomes infested [5]. Traditionally, Fusarium wilt management has relied on chemical fungicides such as benzimidazoles and triazoles, as well as the development of resistant tomato cultivars [5].

However, fungicide-based management strategies present several limitations, including environmental contamination, health risks associated with chemical residues, toxicity to non-target organisms, and the emergence of fungicide-resistant Fusarium strains [6]. Consequently, regulatory authorities, particularly in Europe, have increasingly restricted the use of chemicals because of such concerns [7]. Furthermore, FOL comprises several pathogenic races that can bypass single-gene resistance in tomatoes, with new races still emerging [4]. Therefore, sustainable and effective alternatives for managing Fusarium wilt are urgently needed.

In this context, plant-derived essential oils (EOs) have attracted considerable attention as natural antifungal agents. These EOs are complex mixtures of volatile secondary metabolites produced by aromatic plants and are typically composed of terpenoids, phenolics, and other bioactive compounds [6]. Many EOs exhibit broad-spectrum antimicrobial activity and low environmental persistence, making them attractive candidates for environmentally sustainable crop protection [8]. Phenolic monoterpenes, such as thymol, eugenol, and carvacrol, can disrupt fungal cell membranes, resulting in leakage of intracellular contents and inhibition of enzymes involved in cell wall biosynthesis. These multisite mechanisms reduce the likelihood of resistance development in fungal pathogens. In addition, EOs are generally biodegradable and exhibit relatively low toxicity to mammals [9,10].

Despite these advantages, the direct application of EOs for crop protection is associated with several limitations. Most EOs are hydrophobic and poorly soluble in water, which complicates their application as sprays or soil drenches [11]. Moreover, their high volatility and susceptibility to degradation under field conditions (e.g., UV light, oxygen) reduce their persistence and efficacy [12]. In addition, certain EOs may exhibit phytotoxic effects at high concentrations or possess strong odors, which limit their practical applications [13].

Recently, several formulation strategies have been investigated to overcome these limitations. One major advancement is the application of nanotechnology to develop EO-based nanofungicides [14]. Encapsulating or emulsifying EOs into nanoscale carriers, such as nanoemulsions, polymer nanoparticles, or liposomes, can enhance their stability, enable controlled release, and improve their antifungal efficacy [14].

Nanoparticles, independent of EOs, represent a novel class of antifungal agents for plant disease management. Metallic and metal-oxide nanoparticles (e.g., silver, copper oxide, zinc oxide, and iron oxide) exhibit intrinsic antifungal and antibacterial properties owing to their nanoscale size and high surface reactivity [15,16,17]. These nanoparticles can attach to microbial cell walls and membranes, generate reactive oxygen species, and release metal ions that disrupt essential microbial processes [18]. For instance, silver nanoparticles (AgNPs) have been widely studied for their potent antimicrobial activity and relatively low propensity to select for resistance in target organisms [19]. In plant pathology, a new paradigm is emerging: “nano-biopesticides,” in which either biological compounds (such as EOs or plant extracts) are delivered via nanocarriers, or biologically synthesized nanoparticles themselves function as the active antifungal agents.

Despite the growing number of studies evaluating EOs and nanoformulations against FOL, the available evidence remains fragmented, with limited comparative synthesis across formulations, chemotypes, and modes of action. In particular, the added value of nanotechnology in enhancing antifungal efficacy, formulation stability, and plant protection has not been systematically evaluated. This review addresses this gap by critically synthesizing recent evidence on both conventional and nanomaterial-formulated EO-based strategies for managing Fusarium wilt in tomatoes.

This systematic review synthesizes evidence published between 2000 and 2025 on the use of EOs and nanoformulations to control Fusarium wilt of tomato. This review focuses on studies that evaluate EOs (alone or in conventional formulations) and various nano-based delivery systems against FOL in in vitro or in vivo experimental systems. Specifically, this review identifies the most effective EOs and EO-derived compounds, evaluates the effectiveness of nanoformulations in reducing disease severity, and compares nano-based approaches to unformulated EOs in terms of antifungal efficacy and plant protection. Overall, this review highlights current innovations and research gaps in eco-friendly management of FOL, guiding future research and the potential integration of EO-based fungicides into tomato wilt management strategies.

## 2. Results

The included studies were categorized into two groups for synthesis: (1) studies evaluating EOs and their constituents against FOL and (2) studies assessing EO-based nanoformulations against FOL. Evidence from both in vitro and in vivo studies was considered within each group. Due to the substantial heterogeneity in experimental methodologies and outcome metrics, a quantitative meta-analysis was not feasible; therefore, a descriptive synthesis was conducted. Representative studies in each group are summarized in tables that describe the formulation type, experimental design, and key findings on antifungal activity. The narrative synthesis highlighted recurring findings, including the most effective EOs and nano systems, observed dose–response relationships, improvements associated with nanoformulation, and outcomes reported in plant-based experiments. Publication trends and the efficacy of these treatments relative to conventional fungicides or untreated controls were also evaluated.

### 2.1. Overview of Included Studies

Forty studies met the predefined inclusion criteria. Among these, 31 studies conducted in vitro experiments to evaluate the antifungal activity of EOs, individual EO constituents, vapors, or EO-based nanoformulations against FOL under laboratory conditions. In addition, 21 studies included in vivo or greenhouse evaluations, assessing disease suppression, plant defense activation, soil or seed treatments, and growth and physiological responses in tomato plants challenged with FOL. Furthermore, 16 studies combined both in vitro and in vivo approaches, integrating laboratory assays with pot or greenhouse experiments to validate antifungal efficacy and confirm disease suppression at the plant level.

**Figure 1:**
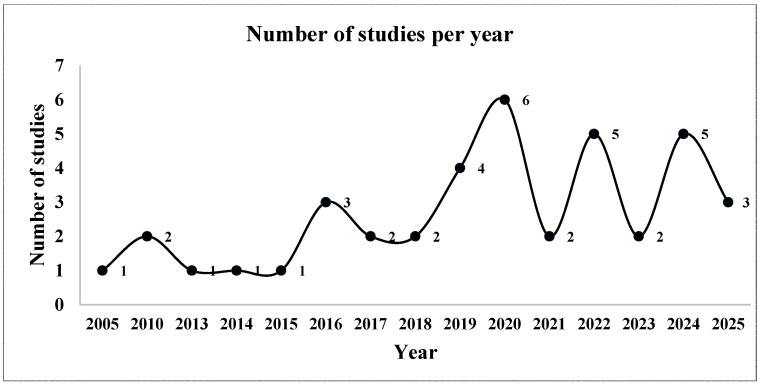
Publication trends of studies included in the systematic review (2000–2025).

The included studies were published between 2005 and 2025 (Figure 1). Research output increased markedly after 2019, with publication peaks observed in 2020, 2022, and 2024, indicating a growing scientific interest in EO-based antifungal strategies. Most studies were conducted in Asia, Africa, and the Mediterranean, with India, Brazil, Egypt, and Mexico as the leading contributors. This geographic distribution reflects the agronomic importance of Fusarium wilt in warm-climate regions, where tomato production is particularly vulnerable to soil-borne pathogens.

### 2.2. In Vitro Efficacy of EOs Against FOL

Across the compiled literature, 51 plant taxa from 21 botanical families were evaluated for their antifungal activity against FOL (Table 1). The evidence base was unevenly distributed across plant families, with Lamiaceae the most dominant, accounting for the largest proportion of the taxa tested. This was followed, at a considerable distance, by Myrtaceae, Apiaceae, Rutaceae, Poaceae, and Brassicaceae. In contrast, several families (e.g., Schisandraceae, Sapotaceae, Simmondsiaceae, and Pedaliaceae) were represented by a single species each.

This taxonomic bias reflects the historical research focus on aromatic plant families and their widespread availability as a source of EOs. Families such as Lamiaceae and Myrtaceae are well known for their high content of volatile secondary metabolites, which have led to their frequent selection as candidate sources for antifungal screening against soil-borne pathogens, including FOL [37,38].

A comparative synthesis of the included studies reveals a clear stratification of plant EOs by antifungal performance against FOL, highlighting significant differences in efficacy, consistency, and translational relevance (Table 2). Among the 51 plant taxa, seven EOs (14%) consistently demonstrated strong efficacy in both laboratory and plant-based experiments.

According to the reviewed studies, EOs demonstrated substantial variability in antifungal potency against FOL. Reported MIC values ranged from 0.05 to 2.0 µL mL^−1^, depending on the plant species, EO chemical composition, and experimental conditions. In vitro assays showed that mycelial growth inhibition typically ranged from 50% to 100% at higher EO concentrations.

Greenhouse and pot experiments further demonstrated that EO treatments can significantly reduce Fusarium wilt severity. Across the reviewed studies, disease suppression generally ranged from 30% to over 70% relative to untreated controls, although the degree of suppression varied depending on application method, concentration, and experimental conditions. In several instances, nanoformulated EOs commonly exhibited enhanced antifungal performance compared with non-formulated oil, indicating improved stability and delivery of bioactive compounds.

Among the evaluated taxa, EOs from *Mentha spicata*, *Mentha longifolia*, *Foeniculum vulgare*, *Syzygium aromaticum*, *Melaleuca alternifolia*, *Lippia berlandieri*, and *Citrus sinensis* demonstrated the most consistent efficacy across both in vitro and in vivo systems. These EOs reduced Fusarium wilt severity by ≥75% and inhibited fungal growth by ≥90% [23,31,34,37,38] indicating strong fungicidal activity without negatively affecting plant growth, supporting their use in advanced formulations and field applications.

In contrast, several EOs, including *Cinnamomum verum* and *Thymus vulgaris*, demonstrated significant antifungal activity in vitro but showed inconsistent results in vivo. This difference is likely due to physicochemical limitations, including volatility, rapid breakdown, short soil persistence, and poor penetration into the root zone, highlighting the need for improved delivery methods [28,29,33,51].

Other EOs, including those from *Allium cepa*, *Artemisia absinthium*, *Aloe vera*, *Eucalyptus globulus*, *Origanum vulgare*, *Citrus limon*, *Cuminum cyminum*, *Cupressus sempervirens*, *Eruca sativa*, *Ocimum gratissimum*, and *Rosmarinus officinalis*, generally achieved a moderate level of disease suppression (25–60%). Although these EOs are not fully effective, their moderate activity suggests potential as supplementary agents in IPM, especially when combined with other treatments or EO mixtures [18,21,35,42].

A distinct group of EOs exhibited antifungal activity primarily via vapor-phase or fumigant mechanisms. Oils from *Mentha spicata*, *Cymbopogon citratus*, *Melaleuca alternifolia*, and *Sideritis germanicopolitana* showed strong anti-fungal properties when used as volatilomes, highlighting the potential of fumigation-based delivery systems for controlling soil-borne pathogens in protected cultivation systems [31,34].

Some EOs appeared to provide plant-mediated rather than strong direct antifungal activity. The use of *Origanum vulgare* subsp. *hirtum*, *Foeniculum vulgare*, *Rosmarinus officinalis*, and *Salvia officinalis* was associated with lower wilt severity due to physiological priming, soil effects, and increased host tolerance [21,24,38].

In contrast, EOs from *Argania spinosa*, *Piper nigrum*, *Moringa oleifera*, *Ocimum selloi*, *Simmondsia chinensis*, and *Capsicum annuum* exhibited limited, inconsistent, or negligible efficacy, while others proved ineffective or had antagonistic effects, such as *Cymbopogon winterianus*, *Salvia hispanica*, and *Satureja horvatii* [22,26,34]. These findings emphasize the importance of basing decisions on robust evidence and caution against presuming antifungal properties solely because of botanical origin or traditional usage.

Phytotoxicity at high doses was also reported for certain other effective EOs, particularly *Syzygium aromaticum*, *Origanum vulgare*, and *Capsicum annuum* [21,28,46]. Detrimental disease suppression at concentrations that affect plant health highlights the critical need to optimize the dose. These results reinforce the prevailing consensus that advanced formulation technologies, especially encapsulation and controlled-release systems, are integral to achieving the best efficacy while safeguarding crop integrity.

In vitro assays showed that the antifungal potency of EOs is intricately linked to their chemical profiles. EOs with high levels of phenolic monoterpenes and oxygenated terpenoids, particularly those from *Lippia berlandieri*, *Origanum vulgare*, *O. vulgare* subsp. *hirtum*, *Trachyspermum ammi*, and *Thymus vulgaris* (rich in carvacrol or thymol) consistently caused almost complete inhibition of FOL at low concentrations [27,44,48,49].

Similarly, citral-rich EOs from *Cymbopogon citratus* and anethole-dominated oils from *Foeniculum vulgare* showed strong fungicidal or fungistatic effects in agar- and broth-based assays, often accompanied by pronounced suppression of sporulation and mycelial development [23,33,36].

Moderate to strong in vitro activity has also been reported for EOs from *Mentha spicata*, *Mentha longifolia*, *Syzygium aromaticum*, *Artemisia absinthium*, and *Cinnamomum* spp., with several studies documenting dose-dependent inhibition and fungicidal effects at higher concentrations [24,35,37,47]. In contrast, EOs dominated by monoterpene hydrocarbons, such as those from *Citrus limon* and *Citrus sinensis*, or by non-volatile lipid fractions, such as *Simmondsia chinensis,* generally showed weaker and less consistent antifungal activity [22,23].

Beyond direct-contact assays, several studies have proved that EOs retain their antifungal activity when applied in the vapor phase, highlighting the contribution of volatile constituents. Vapor-phase or olfactory chamber assays have shown strong inhibition of FOL growth by EOs from *Mentha* spp., *Melaleuca alternifolia*, *Lavandula stoechas*, and *Sideritis germanicopolitana*, even in the absence of direct physical contact with the fungal culture [31,34].

This observation is particularly relevant for soilborne pathogens such as FOL, where volatile diffusion may enable EO activity beyond the immediate site of application. However, vapor-phase efficacy was not universal across all oils, indicating that volatility alone is insufficient and must be considered alongside EO composition. Moreover, results obtained under controlled laboratory conditions may not fully reflect field realities, as factors such as soil absorption, microbial degradation volatilization, and limited diffusion in the rhizosphere can reduce the persistence and bioavailability of EO constituents [27,29,34]. Consequently, EOs that exhibit strong antifungal activity in vitro may show reduced or variable performance under greenhouse or field conditions, underscoring the importance of validating their efficacy in vivo studies.

Across many studies, antifungal responses frequently exhibited a concentration-dependent transition from fungistatic to fungicidal activity, with lower EO doses primarily suppressing mycelial growth, whereas higher concentrations caused complete inhibition or irreversible cellular damage in the pathogen. This pattern was reported for several EOs including those from *Mentha longifolia*, *Cymbopaogon citratus*, *Origanum vulgare*, and *Syzygium aromaticum*, which exhibited dose-dependent inhibition and fungicidal activity at elevated concentrations [32,36,38,41].

In addition to their activity against FOL, many EOs exhibit broad-spectrum antimicrobial properties, enabling them to suppress multiple pathogens simultaneously. Several EO constituents have demonstrated inhibitory effects against a range of fungal phytopathogens, including *Alternaria* and *Botrytis* species [52,53,54]. The multiple target activity may provide synergistic benefits in agricultural systems where crops are frequently exposed to complex pathogen communities or disease complexes, highlighting the potential of EO-based formulations as versatile tools in IPM strategies [55].

### 2.3. In Vivo and Greenhouse Efficacy Against Fusarium Wilt of Tomato

Sixteen of the included studies extended their testing to greenhouse or growth chamber experiments with FOL-inoculated tomato plants, and a few conducted field trials in infested soil.

The in vivo and greenhouse results, summarized in Table 1, showed that a subset of EOs with strong in vitro activity also significantly suppressed Fusarium wilt in tomato plants challenged with FOL. In vivo results were more variable than in vitro, but several EOs significantly reduced Fusarium wilt severity and incidence in tomato. Generally, EOs had to be applied preventively or at the onset of infection to be effective, often as soil drenches or seed treatments.

EOs from *Origanum spp.*, *Foeniculum vulgare*, *Mentha spicata*, *Mentha longifolia*, *Cymbopogon citratus*, and *Cymbopogon winterianus* consistently reduced disease incidence, disease severity, and area under the disease progress curve (AUDPC) under greenhouse or pot conditions [27,36]. EOs from *Origanum spp.*, *Foeniculum vulgare*, *Mentha spicata*, *Mentha longifolia*, *Cymbopogon citratus*, and *Cymbopogon winterianus* consistently reduced disease incidence, disease severity, and AUDPC under greenhouse or pot conditions [20,27,36,37,56]. In several studies, EO treatments achieved levels of disease control comparable to commercial fungicides, with no visible phytotoxic effects [38,56].

Application methods included soil drenching, seed coating or priming, vapor-phase exposure, and EO-rich soil amendments, with soil-based applications being the most frequently reported [27,36]. In addition to disease suppression, several studies documented improved plant growth parameters, including plant height, biomass, chlorophyll content, and yield, in EO-treated plants compared with infected controls [37,56].

Contrary to this, EOs such as *Citrus limon*, *Azadirachta indica*, *Sesamum indicum*, *Salvia hispanica*, and *Piper nigrum* showed restricted or incompatible disease suppression, consistent with their comparatively ineffective in vitro activity [21,23]. These findings highlight that in vitro antifungal efficacy does not always translate directly into effective disease control under greenhouse or field conditions, emphasizing the importance of evaluating EO treatment under realistic plant-pathogen interaction environments.

EOs’ efficacy may also be influenced by the variability of chemical composition associated with plant chemotype, geographic origin, climate conditions, and extraction methods [57,58,59,60] which can alter the relative abundance of key antifungal constituents. Consequently, differences in EO composition may contribute to variability in antifungal performance reported across studies and regions, highlighting the importance of detailed phytochemical profiling and chemical standardization when evaluating EO-based disease management strategies.

### 2.4. Antifungal Activity of EO Compounds

Several studies have evaluated the antifungal activity of individual EO constituents against FOL (Table 3), either as pure compounds or in comparison with their whole EOs. The most frequently investigated compounds belong to monoterpenoids, phenylpropanoids, and sesquiterpenes, including citral, carvacrol, cinnamaldehyde, geraniol, eugenol, citronellol, citronellal, camphor, camphene, carvone, thymol, nerol, trans-anethole, and β-caryophyllene. At the same time, α-pinene and linalool have generally shown limited or inconsistent activity [25,27,42,56]. In many cases, isolated compounds exhibited antifungal potency equal to or greater than that of whole EOs, reflecting their direct interaction with fungal cellular targets; however, high volatility and dose-dependent phytotoxicity remain important constraints for practical application [27,30].

Among the evaluated compounds, oxygenated monoterpenes and aldehydes were consistently associated with the strongest antifungal activity. Citral (neral and geranial) consistently ranked among the most potent compounds, exhibiting fungicidal properties with MIC and MFC values of approximately 512 µg·mL^−1^ and achieving complete mycelial inhibition at concentrations ≥500 µg·mL^−1^ or ≥1.5% (*v*/*v*) [33,61,62]. Consistent antifungal effectiveness was proved by significant radial growth inhibition (84% or more) over a broad range of concentrations [62]. Likewise, geraniol exhibited pronounced antifungal activity, with low IC_50_ values (0.14 µL·mL^−1^) and complete inhibition at concentrations ≥0.5 µL·mL^−1^, underscoring its fungicidal potential [56,62].

Phenolic monoterpenes and phenylpropanoids also demonstrated strong antifungal effects. Carvacrol completely inhibited mycelial growth at 166 µg·mL^−1^, although it exhibited primarily fungistatic activity at lower concentrations [27]. Similarly, thymol fully blocked conidial germination across all tested concentrations, with EC_50_ near 295 µg mL^−1^ [42]. Among cinnamon-derived phenylpropanoids, cinnamaldehyde was the most potent compound, providing high inhibition at relatively low doses, whereas cinnamyl acetate showed only moderate mycelial inhibition [30]. Eugenol had moderate to strong antifungal effects, mainly acting fungistatically and inhibiting mycelial and conidial growth [27,42].

Other oxygenated monoterpenes, such as Citronellol, citronellal, and nerol, also showed measurable antifungal effects. Citronellol demonstrated greater fungicidal activity than citronellal, achieving full inhibition at ≥ 0.5 µL mL^−1^, unlike citronellal’s moderate, mostly fungistatic effect [56]. Nerol completely inhibited FOL at ≥1.5% (*v*/*v*) [33].

Additional compounds, including camphene, camphor, carvone, trans-anethole, and β-caryophyllene, showed moderate antifungal activity, with MIC or IC_50_ values typically between 0.11 and 0.16 mg mL^−1^ [25,39,51]. Contrary to this, -pinene showed no mycelial growth inhibition and only partial suppression of conidial germination, while linalool exhibited antifungal activity without reported quantitative MIC or MFC values against FOL [42,64].

Evidence from in vivo and greenhouse studies, though more limited, has demonstrated the disease-suppressive potential of selected EO-derived compounds. Citral soil drench treatments consistently reduced Fusarium wilt severity by approximately 46–54% and were associated with strong induction of plant defense responses, including chitinase, β-1,3-glucanase, and thaumatin-like proteins [61,63]. Geraniol, applied as a soil treatment, provided wilt suppression comparable to that of a commercial fungicide without visible phytotoxic effects [56]. In addition, carvacrol seed treatments significantly reduced wilt incidence and AUDPC values while supporting seed germination and seedling vigor [27]. Although cinnamaldehyde contributed to disease reduction when delivered as a nanoemulsion, most other compounds have not yet been confirmed under greenhouse or field conditions [30].

### 2.5. Antifungal Efficacy of Nanoformulated EOs and EO Compounds

Several studies have explored nano-delivery and controlled-release systems to enhance the antifungal performance of EOs and their major bioactive compounds against FOL (Table 4). The main formulation approaches include polymeric nanoparticles, nanoemulsions, cyclodextrin inclusion complexes, and volatilome-based phyto-fumigation systems, each aimed at improving EO stability, dispersion, and controlled release.

The effectiveness of nanoformulation strategies, however, varies considerably depending on encapsulation efficiency, release kinetics, and compatibility between the active compound and carrier system. For example, citral encapsulated in chitosan nanoparticles had much lower antifungal activity than free citral, with mycelial inhibition below 27.2% and an MIC of 4096 µg mL^−1^, likely due to poor encapsulation and slow release [61]. In contrast, free citral completely inhibited mycelial growth at 500–1000 µg mL^−1^ with MIC and MFC values around 512 µg mL^−1^ and reduced Fusarium wilt severity by 44–53% in greenhouse tests and soil drench experiments [61]. This example illustrates that nanoformulation does not always enhance bioactivity, and the formulation design must be carefully optimized.

Among the tested systems, nanoemulsion systems enhance EO effectiveness. A botanical nanoemulsion with cinnamaldehyde-rich cinnamon EO and *Annona squamosa* seed extract showed strong in vitro inhibition (91.6% at 1:100 dilution) and disease control rates of 49.6–57.1% in fruit assays, showing better dispersion and bioavailability than bulk oils [30]. Similarly, non-ionic nanoemulsions combining clove and lemongrass EOs reduced MIC from 7000 mg L^−1^ for free EO to 4000 mg L^−1^ and achieved up to 70.6% wilt reduction without phytotoxicity under greenhouse conditions [65]. Nanoemulsified clove EO with droplet sizes of 20–50 nm also showed complete inhibition of *F. oxysporum* at lower doses than free EO, confirming the benefits of nanoscale dispersion [32].

Cyclodextrin-based encapsulation has also shown promising results, improving EO stability and prolonging release. β-Cyclodextrin inclusion complexes of clove EO increased antifungal inhibition from ≤6.3% to 70.1% at 400 ppm in liquid assays [47]. Similarly, β-cyclodextrin microcapsules improved the inhibition zone for clove EO and Mexican oregano EO (*Lippia berlandieri*), showing prolonged release and improved persistence compared with non-encapsulated EOs [46].

Alternative delivery systems based on volatile organic compounds (volatilomes) have also been explored. Volatile compounds from Mentha spicata, immobilized in vermiculite balls, achieved 92.4% mycelial growth inhibition and reduced wilt incidence to 8.3% compared with complete disease development in untreated controls, demonstrating highly effective vapor-phase delivery under greenhouse conditions [31]. By contrast, volatilomes from *Cymbopogon citratus* showed weaker antifungal effects (75.3% inhibition) with no reported in vivo validation [31].

**Figure 2:**
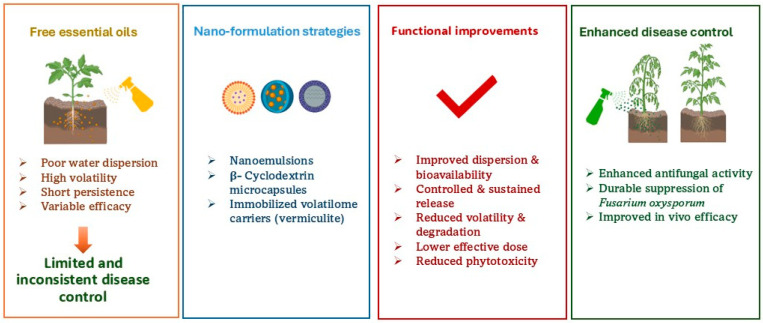
Role of nanoformulation in improving essential oil-based control of tomato Fusarium wilt.

Overall, the effectiveness of nano-delivery systems against FOL appears highly dependent on both compound chemistry and formulation strategy. While some formulations (e.g., nano-citral with low loading efficiency) may reduce antifungal activity, others, including nanoemulsions, cyclodextrin complexes, and volatilome-based systems, generally enhance dispersion, stability, and biological performance compared to free EOs (Figure 2).

Despite these promising findings, the practical application of nanoformulated EO-based antifungal agents in agriculture remains limited by several constraints. Large-scale production of stable nanoformulations may involve higher manufacturing costs and specialized formulation technologies, which could affect economic feasibility for agricultural use [66,67]. In addition, regulatory frameworks governing nano-enabled crop protection products require comprehensive environmental and toxicological evaluation, particularly regarding potential impacts on non-target organisms and soil ecosystems [68,69]. Therefore, further research integrating field-scale validation, environmental safety assessment, and economic analysis will be essential to determine the practical feasibility of nanoformulated EO-based strategies for sustainable management of Fusarium wilt in the tomato production system.

### 2.6. Mechanistic and Molecular Basis of EO Activity Against FOL

The mechanisms underlying the antifungal activity of EOs against FOL have been investigated in several experimental studies; however, the level of evidence supporting different mechanisms varies among the reviewed literature. In this review, a distinction is made between mechanisms that have been experimentally demonstrated, such as membrane disruption, increased membrane permeability, and leakage of intracellular components observed through microscopy and biochemical assays, and mechanisms that are proposed based on indirect evidence or analogy with other fungal pathogens.

**Figure 3:**
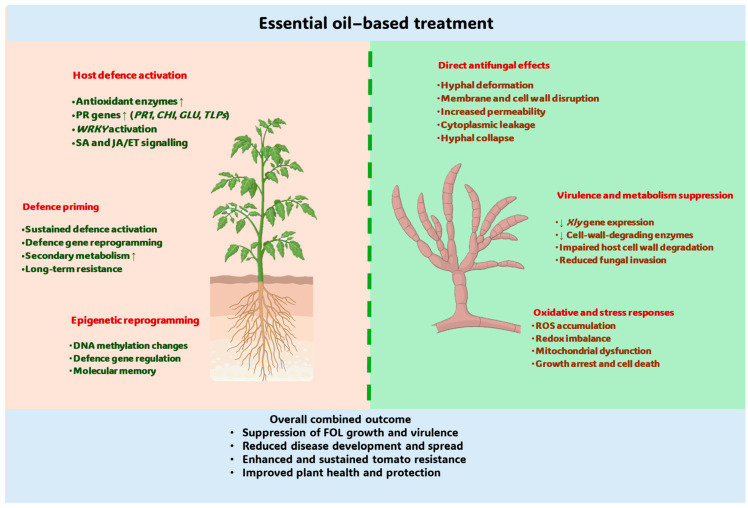
Direct antifungal activity and host-mediated defense priming induced by essential oil application. Upward (↑) and downward (↓) arrows indicate increases and decreases, respectively, in gene expression and associated biological processes.

Collectively, the available evidence shows that EOs and their bioactive constituents suppress FOL through multilevel mechanisms, including direct anti-fungal effects on fungi and indirect activation of host plant defense responses (Figure 3). However, the level of evidence supporting these mechanisms varies across studies. Several mechanisms, including membrane disruption, increased membrane permeability, cytoplasmic leakage, and inhibition of mycelial growth, have been experimentally demonstrated using microscopy, physiological assays, and biochemical analyses. In contrast, other proposed mechanisms, including interference with fungal metabolic pathways, inhibition of enzymes involved in cell wall synthesis, and modulation of fungal gene expression or host defense signaling, are inferred from indirect observation or extrapolated from related plant-pathogen systems. These mechanisms collectively span cellular, biochemical, molecular, and epigenetic routes, together causing disease suppression in tomato [23,24,27,36].

#### 2.6.1. Direct Antifungal Effects on FOL Structure and Physiology

At the cellular level, EOs rich in phenolic monoterpenes and oxygenated terpenoids (e.g., carvacrol, thymol, citral, eugenol, cinnamaldehyde) cause noteworthy damage to FOL. These EOs induce the destruction of fungal hyphae, cell membrane breakdown, cytoplasmic leakage, vacuole formation, and fungal cell collapse, showing compromised cell wall and membrane integrity [23,36,70]. This leads to dose-dependent inhibition of mycelial growth, conidial germination, and sporulation, with fungistatic or fungicidal effects seen in laboratory assays [23,27,44]. Vapor phase assays also prove that EO volatilomes inhibit fungal growth via volatile constituents without direct contact [31,34].

#### 2.6.2. Suppression of FOL Virulence and Metabolism at the Molecular Level

Treatments of plants and FOL with EOs not only cause physical damage to FOL but also decrease fungal pathogenicity by down-regulating xylanase (*Xly*) genes, which code for essential cell-wall-degrading enzymes necessary for vascular penetration. This leads to lower xylanase activity and decreases fungal invasiveness [35]. Similarly, citral and citronella-derived compounds decrease cellulase activity and/or expression, harming the degradation of plant cell walls and inhibiting fungal spread within host tissues [23,56]. Together, these findings show that EO activity extends beyond growth inhibition to include targeted disruption of virulence-associated functions [23,35].

#### 2.6.3. Induction of Oxidative and Stress-Response Pathways in Fungal Cells

Exposure to EOs may trigger oxidative stress in FOL. Elevated levels of reactive oxygen species (ROS) and disruption of redox balance have been reported following treatment with Mentha, Origanum, and Cymbopogon EOs, suggesting impairment of energy metabolism and cellular respiration [37]. Transcriptomic and biochemical evidence further show that fungal antioxidant defense systems may be insufficient to counteract EO-induced ROS accumulation, contributing to growth arrest and cell death [24,37]. These observations support a multi-target stress-based mode of antifungal action at the molecular level [37].

#### 2.6.4. Activation of Tomato Defense-Related Physiological and Molecular Responses

In vivo and greenhouse studies consistently show that EO application activates tomato defense mechanisms. EO treatments increase activities of antioxidant enzymes, including superoxide dismutase (SOD), catalase (CAT), and ascorbate peroxidase (APX), and promote the accumulation of phenolic and flavonoid compounds [36,37]. At the transcriptional level, EO exposure induces pathogenesis-related (*PR*) genes, including *PR1*, chitinase (*CHI*), β-1,3-glucanase (*GLU*), and thaumatin-like proteins (*TLPs*) [37]. In parallel, *WRKY* transcription factors are upregulated, coordinating downstream defense networks [36,37]. These transcriptional changes have been associated with activation of both salicylic acid (SA) and jasmonic acid/ethylene (JA/ET) signaling pathways, consistent with broad-spectrum immune activation in tomato following EO treatment [23].

#### 2.6.5. Defense Priming and Epigenetic Reprogramming in the Host

Advanced molecular investigations show that certain EOs can induce defense priming in tomato plants. For example, *Artemisia absinthium* EO has been linked to epigenetic modifications, including changes in DNA methylation patterns, accompanied by transcriptional reprogramming of genes involved in defense, secondary metabolism, and stress tolerance [24]. Metabolomic and transcriptomic studies show coordinated regulation of phenylpropanoid, flavonoid, and lignin biosynthesis, leading to cell wall strengthening and increased antimicrobial compounds [24]. These molecular changes can persist after treatment, indicating EO-induced molecular memory [24].

#### 2.6.6. Modulation of Mechanisms by Nanoformulation

Nanoformulation of EOs and EO compounds influences antifungal mechanisms by changing bioavailability, stability, and release kinetics. Cyclodextrin inclusion complexes have been reported to enhance molecular interactions between active compounds (e.g., eugenol) and fungal targets, leading to stronger inhibition of gene expression linked to growth and virulence [47]. Conversely, polymeric nanoparticle systems, such as citral-loaded chitosan nanoparticles, exhibit delayed molecular effects consistent with controlled release, with reduced immediate transcriptional suppression and prolonged activity over time. These findings highlight the importance of formulation-driven molecular dynamics in controlling antifungal performance [47,61].

Together, the evidence indicates that EOs suppress FOL through integrated cellular disruption, suppression of virulence-associated enzymes/genes, stress induction, and host defense activation and priming, with outcomes shaped by EO chemistry, delivery mode, and formulation design [23,24,27,36,47]. Overall, the antifungal efficacy of EOs against FOL is governed by EO chemistry, delivery strategy, and plant pathogen context. Phenolic and aldehyde-rich EOs consistently showed the strongest activity, while nanoformulation appeared as a key tool to enhance stability and field relevance. These findings support the integration of EO-based nano-biopesticides into sustainable Fusarium wilt management strategies.

## 3. Materials and Methods

### 3.1. Literature Search Strategy

Following the PRISMA 2020 guidelines [71], we reviewed the literature from January 2000 to November 2025 using the Web of Science, PubMed, ResearchGate, and Google Scholar. The review was conducted in October 2025 and updated through November 2025, using keywords related to the pathogen, interventions, and crop (for example, “*Fusarium oxysporum* f.sp. *lycopersici*”, “Fusarium wilt tomato”, “essential oil”, specific oil names, “nano”, and relevant nanomaterial terms) with “tomato” or “*Solanum lycopersicum*”. Furthermore, we manually checked the reference lists of key review articles. We focused on peer-reviewed literature and did not search gray literature (e.g., dissertations or technical reports), prioritizing studies with accessible full-text and robust data. The output was also imported into reference management software (Mendeley, Version 2.143.0, Elsevier Ltd., London, UK) and screened for duplicate references.

### 3.2. Inclusion and Exclusion Criteria

**Figure 4:**
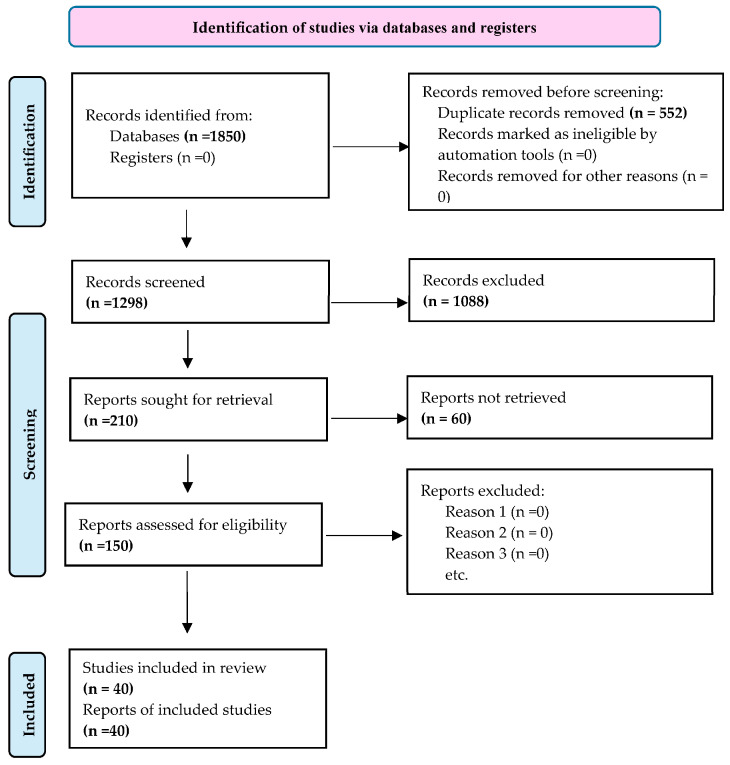
Flowchart of studies selected for the systematic review according to the PRISMA criteria (2020).

To ensure relevance and consistency of the studies included in this systematic review, predefined inclusion and exclusion criteria were applied during the screening process. The study selection process followed the PRISMA 2020 guidelines and consisted of four stages: identification, screening, eligibility assessment, and final inclusion (Figure 4). Titles and abstracts were initially screened to remove irrelevant records. The full text of potentially relevant studies was subsequently evaluated against the predefined inclusion and exclusion criteria.

Studies were considered eligible for inclusion if they met the following criteria: (i) peer-reviewed research articles published between January 2000 and November 2025; (ii) studies evaluating the antifungal activity of plant-derived EO, their major constituents, or EO-based nanoformulations; (iii) studies targeting FOL, the causal agent of Fusarium wilt in tomato; (iv) studies reporting antifungal activity in vitro or assessing disease suppression in vivo under greenhouse, pot or plant-based experimental conditions; and (v) studies providing measurable outcomes related to fungal growth inhibition, disease incidence or severity reduction, or plant physiological or molecular defense responses.

Studies were excluded if they met any of the following conditions: (i) the target pathogen was not FOL or the host plant was not tomato; (ii) the tested material consisted of plant extracts, crude phytochemicals, or synthetic fungicides without the involvement of EOs or EO-derived compounds; (iii) the publication type corresponded to review articles, conference abstracts, book chapters, editorials or short communications lacking original experimental data; or (iv) the full text was not accessible or the methodological description was inadequate to evaluate the antifungal outcomes.

### 3.3. Study Selection Process

A total of 1850 records were identified through the database search. After removing 552 duplicates, 1298 records remained for title and abstract screening. During this stage, 1088 records were excluded because they were unrelated to tomato Fusarium wilt, did not involve EOs or nanoformulations, focused on other pathogens, or represented non-primary literature such as reviews or conference abstracts.

Following initial screening, 210 studies were retained for full-text evaluation. Following assessment against the inclusion and exclusion criteria, 110 studies were excluded because they did not specifically target FOL, evaluated plant extracts rather than EOs, or lacked sufficient experimental data on antifungal efficacy.

Of the remaining 100 eligible studies, 60 articles were further excluded due to incomplete methodological descriptions, lack of quantitative anti-fungal results, or absence of accessible full texts. Ultimately, 40 studies met all inclusion criteria and were included in the qualitative synthesis. The study selection workflow is illustrated in the PRISMA flowchart (Figure 4).

### 3.4. Data Extraction

Data from the selected studies were systematically extracted and organized into structured tables to facilitate comparison and synthesis of the findings. The extracted information included: author and year of publication of plant species used for EO extraction, major chemical constituents of the EO, formulation type (pure EO, nanoemulsion, nanoparticle-based formulation, or other nano-delivery systems), experimental conditions (in vitro or in vivo/greenhouse experiments), pathogen strain, and reported antifungal outcomes.

Outcome measures extracted from the studies included percentage inhibition of fungal mycelial growth, MIC, reductions in disease incidence or severity in tomato plants, and observed physiological or molecular plant responses associated with disease resistance.

During data extraction, reported antifungal outcomes were categorized as fungistatic activity, defined as inhibition of fungal growth without complete loss of viability, or fungicidal activity, defined as irreversible fungal cell death resulting in the inability of the pathogen to resume growth after treatment removal.

### 3.5. Study Quality Assessment

To evaluate the methodological reliability of the included studies, a qualitative assessment was conducted using criteria adapted to plant pathology and agricultural experimental studies. Each study was examined based on several methodological indicators, including: (i) clarity of experimental design, (ii) appropriate identification and characterization of FOL, (iii) description of EO composition or chemical characterization, (iv) presence of appropriate control treatments, (v) replication of experiments and statistical analysis, and (vi) reporting of quantitative antifungal outcomes such as percentage growth inhibition, disease incidence, or severity reduction.

Based on these criteria, studies were categorized as high, moderate, or low quality, depending on the completeness of methodological reporting and robustness of experimental procedures. Studies that clearly describe experimental protocols, include proper controls, and provide quantitative antifungal results supported by statistical analysis were considered high quality. Studies lacking some methodological details but still reporting measurable outcomes were classified as moderate quality, whereas studies with insufficient methodological transparency or limited experimental detail were categorized as low quality. The quality assessment was used to support the interpretation of results and identify potential methodological limitations among included studies. Overall, most included studies were classified as moderate to high methodological quality, with most investigations reporting replicated experiments and quantitative antifungal measurements.

### 3.6. Data Synthesis

Due to the heterogeneity in experimental designs, plant species, EO compositions, and evaluation methods across the included studies, a qualitative synthesis approach was adopted rather than a quantitative meta-analysis. The results were therefore summarized and discussed by grouping studies according to the type of EO tested, formulation strategy, and type of experimental evaluation.

All eligible studies were synthesized chronologically and thematically across the 25-year period. Older foundational studies are referenced only sparingly in the context. The quality of evidence in the included studies was discussed in terms of experimental rigor (e.g., whether they included proper controls, replicates, and statistical analyses). The goal is to provide a comprehensive overview of recent progress in this field and to identify a consensus and gaps that merit further investigation.

## 4. Conclusions

This review shows that EOs and their nanoformulations offer eco-friendly solutions for managing Fusarium wilt in tomato. EOs high in phenolics and aldehydes, plus their active ingredients, prove to have strong antifungal effects against FOL, as proven in labs and plant trials. Nanoformulations enhance EO stability and effectiveness but require further optimization. Future studies should emphasize standardizing tests, confirming mechanisms in field settings, and examining impacts on soil health and other organisms. Using EO-based nano-biopesticides in disease management could lower reliance on synthetic fungicides and promote sustainable tomato farming.

## Figures and Tables

**Table 1:** Reported in vitro and in vivo effects of plant EOs against FOL.

Plant (Family)	In Vitro Antifungal Activity	In Vivo Efficacy	Reference
*Allium cepa* L. (Amaryllidaceae)	Strong inhibition (71.8%) of FOL mycelial growth	Reduced wilt severity by 67.7%; improved plant performance	[20]
*Allium sativum* L. (Amaryllidaceae)	Moderate to strong activity: inhibition zones of 21.2 mm (25 µL mL^−1^) and 33.8 mm (50 µL mL^−1^); complete inhibition at 100 µL mL^−1^. Also 37.84% inhibition at 500 mg L^−1^; dose-dependent	Soil application (1000 ppm/pot): disease severity index 1.6; 55.52% suppression, superior to fungicide alone. EC_50_ = 52.6 mg kg^−1^ soil	[21,22,23]
*Aloe vera* (L.) Burm.f. (Asphodelaceae)	46.2% inhibition of FOL mycelial growth (lowest among tested oils)	Reduced wilt severity by 55.6% in FOL + *Meloidogyne incognita* complex	[20]
*Argania spinosa* (L.) Skeels (Sapotaceae), Accepted name (*Sideroxylon spinosum* L.)	Weak in vitro activity (≤18.92% inhibition at 500 mg L^−1^)	EC_50_ = 39.2 mg kg^−1^ soil; limited disease suppression	[22]
*Artemisia absinthium* L. (Asteraceae)	Strong fungicidal activity: spore germination inhibition 44.25 ± 1.72% at 0.5 mg mL^−1^; EC_50_ = 109.91 µg mL^−1^	Seed coating reduced disease ratio: improved water status (+15%), reduced fresh weight loss (–30%), enhanced pigments	[24]
*Artemisia annua* L. (Asteraceae)	Strong inhibition (77.16%) of mycelial growth; MIC = 0.22 ± 0.03 mg mL^−1^	Soil amendment reduced disease incidence from 72.22% to 25.00%	[25]
*Azadirachta indica* A. Juss. (Meliaceae)	The inhibition was 27.03% at 250–500 mg L^−1^ and strong (92.82%) at 100 µL mL^−1^EC_50_ = 163 mg kg^−1^ soil. Inhibition zones: 9.2–17.5 mm	Not reported	[22,23,26]
*Brassica nigra* (L.) W.D.J.Koch (Brassicaceae)	Not reported	Disease severity reduced by 38.92%	[21]
*Cinnamomum zeylanicum* Blume (Lauraceae), *Accepted name: Cinnamomum verum* J.Presl	Complete inhibition at 600 µg mL^−1^; EC_50_ = 171.79 µg mL^−1^; EC_100_ = 575.18 µg mL^−1^	Not reported	[27]
*Cinnamomum aromaticum* Nees (Lauraceae), *Synonym: Cinnamomum cassia* (L.) J.Presl	Fungicidal activity only at 10% (*w*/*w*); inactive at lower doses	Not reported	[28]
*Callistemon citrinus* (Curtis) Skeels (Myrtaceae), *Syn: Melaleuca citrina* (Curtis) Dum. Cours.	Complete inhibition at 2500 µg mL^−1^	Not reported	[29]
*Capsicum annuum* L. (Solanaceae)	Low inhibition at 5–10 µL mL^−1^ (17.56–23.08%); high inhibition (92.69%) at 100 µL mL^−1^	Poor disease control even at higher concentrations	[26]
*Chenopodium ambrosioides* L. (Amaranthaceae), *Accepted name: Dysphania ambrosioides* (L.) Mosyakin & Clemants	Complete inhibition at 10 µL mL^−1^; 75.64% at 5 µL mL^−1^	Preventive treatment reduced diseased leaves by 50%, comparable to or superior to fungicides.	[26]
*Cinnamomum cassia* (L.) J.Presl (Lauraceae), *Synonym: Cinnamomum aromaticum* Nees	Very strong inhibition: cinnamon EO and nanoemulsions caused 92% mycelial inhibition at 1% and 57% at 0.5%	Disease reduction 55–57%; non-phytotoxic	[30]
*Citrus limon* (L.) Osbeck (Rutaceae)	Fungicidal activity at 10% (*w*/*w*)	Disease severity was suppressed by 22.16%	[21,28]
*Citrus sinensis* (L.) Osbeck (Rutaceae)	Weak inhibition; 18.92% and 27.03% inhibition at 250 and 500 mg L^−1^, respectively. The largest inhibition zones were 47.5 and 46.3 mm at 25 and 50 µL mL^−1^, respectively. Complete inhibition at 10% (*w*/*w*); inactive at ≤1%	Soil drench EC_50_ = 19.2 mg kg^−1^. Foliar spray: lowest disease severity (PDS = 3.1) at 80 µL mL^−1^; seed treatment gave lowest PDS = 2.11 and disease incidence = 1.5	[22,23,28]
*Cuminum cyminum* L. (Apiaceae)	Inhibition zones: 27.0 mm (50 µL mL^−1^) and 15.3 mm (25 µL mL^−1^)	Seed treatment: PDS = 12.44, PDI = 60, PEDC = 40, disease incidence = 4.5. Foliar spray (60 µL mL^−1^): PDS = 12.0–12.9; moderate disease suppression	[23]
*Cupressus sempervirens* L. (Cupressaceae)	Strong inhibition (82.02%) at 10 µL mL^−1^; moderate inhibition (64.62%) at 5 µL mL^−1^; near-complete inhibition at 20 µL mL^−1^	Preventive application significantly reduced disease and improved growth; the curative effect is limited.	[26]
*Cymbopogon citratus* (DC.) Stapf (Poaceae)	Strong inhibition (75.29%) in the olfactory chamber. MIC = 62.5 ppm, MFC = 250 ppm, and IC_50_ = 24.25 ppm (mycelium) and 0.98 ppm (spores). PDA-amended: up to 100% inhibition at 2.5% (*v*/*v*)	Not evaluated in pots/greenhouse	[31,32,33]
*Cymbopogon winterianus* Jowitt ex Bor (Poaceae), *Common name:* Java citronella	Ineffective under fumigation; increased mycelial growth and sporulation	No disease suppression; seed germination unaffected	[34]
*Eruca sativa* Mill. (Brassicaceae)	Strong inhibition (67.7%) of FOL mycelial growth	Soil treatment reduced wilt severity by 66.0–67.7%	[20]
*Eucalyptus globulus* Labill. (Myrtaceae)	Moderate inhibition (15.9–72.5%); MIC = 500 ppm; IC_50_ = 207.86 ppm. Also, 60.6% inhibition in comparative assays	Reduced wilt severity by 65.3%; improved plant growth	[20,35]
*Eucalyptus grandis* W.Hill ex Maiden (Myrtaceae)	Strong mycelial inhibition; among the most active Eucalyptus leaf EOs	Some reduction in wilt severity in mixed soil-borne disease trials; limited FOL-specific greenhouse data	[23]
*Eucalyptus tereticornis* Sm. (Myrtaceae)	Moderate antifungal activity; complete inhibition at 2500 µg mL^−1^; fungicidal to mycelium and conidia	Not reported	[29]
*Foeniculum vulgare* Mill. (Apiaceae/Umbelliferae)	Strong dose-dependent inhibition: 83% mycelial inhibition and 97% sporulation inhibition at 500 µL mL^−1^ and 44% at 250 µL mL^−1^. IC_50_ = 300.37 µL mL^−1^; complete growth suppression at 500 µL mL^−1^; severe hyphal alterations (lysis, vacuolization, chlamydospore formation)	Curative soil drench (500 µL mL^−1^) reduced disease severity from 98% to 57% at 8 weeks; greenhouse curative treatment reduced severity by 42.85%. Marked improvement in growth, pigments, sugars, phenolics, flavonoids; strong induction of *PR1*, *WRKY*, *TLP*, *LOX*, *ERF*, chitinase, β-1,3-glucanase	[36]
*Lavandula stoechas* L. (Lamiaceae)	Partial hyphal damage (SEM), but continued mycelial growth	Increased mean time to germinate (MTG = 2.48 days); no effective disease suppression	[34]
*Melaleuca alternifolia* (Maiden & Betche) Cheel (Myrtaceae)	Strong mycelial inhibition: SEM revealed hyphal deformation and collapse. Seed-to-PDA assay: complete suppression of mycelial growth (0 cm) for 144 h after seed fumigation (20 µL EO, 24 h)	Seed fumigation reduced seedlings with mycelial growth from 35% to 5% (~80% reduction); abnormal seedlings were reduced (10% vs. 40%); germination was unaffected (≈81–90%); no induction of chitinase or β-1,3-glucanase	[34]
*Mentha × piperita* L. (Lamiaceae)	MIC = 125 ppm; MFC = 500 ppm; IC_50_ = 60.05 ppm (mycelium) and 3.2 ppm (spores)	Not reported	[35]
*Mentha longifolia* (L.) L. (Lamiaceae)	Complete inhibition at ≥1.0% (*v*/*v*); strong fungicidal effect after 7 days	Root rot severity reduced to 3.5%; enhanced plant growth (max height 32.42 cm); increased SOD, CAT, APX activities	[37]
*Mentha spicata* L. (Lamiaceae)	Dose-dependent inhibition (0.25–1.25% *v*/*v*); 92.55% mycelial inhibition at 1.25% *v*/*v*. Volatilomes inhibited FOL by 92.35% (olfactory chamber assay)	EO treatment reduced root rot severity to 5.6% (vs. 86.39%). Soil amendment (4% *w*/*w* plant material) reduced AUDPC from 160 to 51.25. Volatilomes vermiculite balls reduced wilt incidence to 8.33% (91.67% reduction)	[31,37,38]
*Moringa oleifera* Lam. (Moringaceae)	Lower inhibition compared with citrus and mint oils	Weak–moderate protection; not a leading candidate for Fusarium wilt control	[23]
*Ocimum gratissimum* L. (Lamiaceae)	Two response types reported: (i) hyphal damage with persistent sporulation; (ii) complete mycelial inhibition at 625 µg mL^−1^ with fungicidal effect at higher concentrations	Some disease reduction, but consistently lower efficacy than *Melaleuca alternifolia*	[29,34]
*Ocimum selloi* Benth. (Lamiaceae), *Synonym: Ocimum carnosum* (Spreng.) Link & Otto ex Benth.	Hyphal narrowing and curling observed; no growth arrest	No disease suppression	[34]
*Ocimum tenuiflorum* L. (Lamiaceae), *Syn*onym: *Ocimum sanctum* L.	Moderate antifungal activity	Not reported	[39]
*Origanum vulgare* L. (Lamiaceae)	EO caused 61% inhibition at 1% (*w*/*w*) and complete fungicidal activity at 10% (*v*/*v*). PDA assay (4 µL/dish): significant inhibition for all biotypes; Leptokaria biotype showed complete inhibition	Greenhouse: low dose (16 µL/plant) non-phytotoxic and increased yield; high dose (≥97 µL/plant) phytotoxic. Soil application (1000 ppm/pot) under M. incognita + FOL reduced disease severity by 55.52%	[21,28,40]
*Origanum vulgare* subsp. *hirtum* (Link) Ietsw. (Lamiaceae)	Not tested by direct contact; EO-derived soil volatilomes rich in carvacrol (78.31%); volatiles persisted up to 60 DAI	Soil amendment (4% *w*/*w*): AUDPC reduced ~2.6-fold; yield increased 77–95%; Fol symptoms partial, Vs symptoms absent at 50 DAT; chlorophyll +38–62%, photosynthesis +79%	[38]
*Pimenta dioica* (L.) Merr. (Myrtaceae)	Up to 97.78% inhibition of mycelial development within 7.2 days	Not reported	[41]
*Rosmarinus officinalis* L. (Lamiaceae), Accepted name: *Salvia rosmarinus* Spenn.	Weak in vitro inhibition (≤300 µg mL^−1^); limited effect on conidial germination, Moderate antifungal activity	In the greenhouse, the disease severity was reduced by 20.3–35.6% (150–300 µg mL^−1^) and 30.5–47.5% (150–250 µg mL^−1^), indicating an indirect/plant-mediated effect. Greenhouse: 38–39% reduction in wilt severity; partial control	[21,42]
*Piper nigrum* L. (Piperaceae)	Weak activity: Mycelial growth remained high (6.03–7.5 cm at 100–500 ppm). Major compounds: limonene, sabinene, β-caryophyllene	Not effective in vivo; no significant disease suppression on tomato fruit	[43]
*Salvia hispanica* L. (Lamiaceae)	Highest mycelial growth among tested treatments; ineffective	No disease suppression	[34]
*Salvia officinalis* L. (Lamiaceae)	Not evaluated separately in vitro	Disease severity suppression 33.32%; gall suppression 37.16%; egg mass suppression 34.06%	[21]
*Satureja horvatii* Šilić (Lamiaceae)	Essentially, no antifungal activity at tested doses	No meaningful in vivo effect	[44]
*Sesamum indicum* L. (Pedaliaceae)	Not evaluated separately in vitro	Disease severity suppression 22.16%; gall suppression 46.44%; egg mass suppression 49.94%	[21]
*Sideritis germanicopolitana* Bornm. (Lamiaceae)	EO fumigant: 19.71% inhibition (2 µL/Petri), 47.54% (5 µL/Petri). Methanol extract: up to 44.76% inhibition	Not reported	[45]
*Simmondsia chinensis* (Link) C.K.Schneid. (Simmondsiaceae)	Weak inhibition (32.43%) at 500 mg L^−1^	EC_50_ = 43.0 mg kg^−1^ soil	[22]
*Syzygium aromaticum* (L.) Merr. & L.M.Perry (Myrtaceae)	Strong dose-dependent inhibition. PDA: 100% inhibition at ≥500 ppm. MIC 31.25 ppm; IC_50_ = 18.22 ppm (mycelia), 0.3 ppm (spores). Nano-EO improved stability. β-CD encapsulation doubled the inhibition zone	Pot trial: 5% soil emulsion reduced wilt severity by 86.5% (1% = 61.8%); 10% phytotoxic. Postharvest fruit assays showed reduced efficacy vs. in vitro	[35,43,46,47]
*Thymus vulgaris* L. (Lamiaceae)	Complete fungicidal activity at 10% (*w*/*w*). PDA-amended: 44.4% (0.25%), 60.0% (0.5%), 82.2% (1%), 100% (1.5%) inhibition	Not reported	[28,33]
*Trachyspermum ammi* (L.) Sprague (Apiaceae)	Strong inhibition via membrane disruption (thymol-rich EO)	Pot trials: 50% reduction in wilt severity; improved growth	[23,48]
*Lippia berlandieri* Schauer (Verbenaceae)	Extremely potent: MIC 0.2 µL mL^−1^ (contact), 0.15 µL mL^−1^ air (volatile). ≥93% inhibition; biomass fully inhibited	Seed treatment (0.5% EO) completely prevented seed colonization; germination was unaffected.	[49]
*Lavandula dentata* L. (Lamiaceae)	Strong dose-dependent inhibition; ≥1 µL mL^−1^ caused 100% inhibition for all strains	Not evaluated	[50]
*Illicium verum* Hook.f. (Schisandraceae)	Strong activity: IC_50_ = 0.14 mg mL^−1^; trans-anethole identified as principal active compound	Not evaluated	[51]

**Table 2:** Summary of antifungal efficacy patterns of plant-derived EOs against FOL.

Category	Main Findings	Plants Included	Key Implication
Highly effective in vitro and in vivo	Consistent strong antifungal activity (≥90% inhibition and/or ≥75% disease reduction); fungicidal effects and/or strong host protection	*Mentha spicata,*	Top candidates for formulation development, nano-delivery, and field translation
		*Mentha longifolia*	
		*Foeniculum vulgare*	
		*Syzygium aromaticum*	
		*Melaleuca alternifolia*	
		*Lippia berlandieri*	
		*Citrus sinensis*	
Strong in vitro but limited/untested in vivo	Potent mycelial inhibition or fungicidal activity, but no or limited greenhouse/pot validation	*Cinnamomum verum*	Require in vivo validation or improved delivery systems (e.g., nano-encapsulation, slow-release)
		*Thymus vulgaris*	
		*Illicium verum*	
		*Lavandula dentata*	
		*Lavandula stoechas*	
		*Pimenta dioica*	
		*Mentha × piperita*	
		*Cymbopogon citratus*	
		*Eucalyptus tereticornis*	
		*Callistemon citrinus*	
Moderate efficacy (both levels)	Partial inhibition and moderate disease suppression (≈25–60%) under laboratory and greenhouse conditions	*Allium cepa*,	Useful components of IPM strategies, mixtures, or complementary treatments
		*Artemisia absinthium,*	
		*Artemisia annua*,	
		*Aloe vera*,	
		*Eucalyptus globulus*,	
		*Eucalyptus grandis*,	
		*Origanum vulgare*,	
		*Citrus limon*,	
		*Cuminum cyminum*,	
		*Cupressus sempervirens*,	
		*Eruca sativa*,	
		*Ocimum gratissimum*,	
		*Rosmarinus officinalis*	
Volatilomes/fumigation-effective oils	Strong antifungal activity via the vapor phase rather than direct contact	*Mentha spicata*,	Suitable for soil fumigation, seed treatment, volatilome-based, or slow-release systems
		*Cymbopogon citratus*	
		*Melaleuca alternifolia*	
		*Sideritis germanicopolitana*	
Plant-mediated/indirect protection	Modest direct antifungal effect but significant disease reduction via induced resistance, physiological priming, or soil effects	*Origanum vulgare* subsp. *hirtum*,	Indicate defense priming and host-mediated resistance mechanisms
		*Foeniculum vulgare*,	
		*Rosmarinus officinalis*,	
		*Salvia officinalis*	
Weak or inconsistent efficacy	Low inhibition and/or poor or inconsistent disease control	*Argania spinosa*, *annuum*	Low priority for further development against Fusarium wilt
		*Piper nigrum*,	
		*Moringa oleifera*,	
		*Ocimum selloi*	
		*Simmondsia chinensis*,	
		*Capsicum*	
Ineffective or antagonistic	No inhibition or stimulation of fungal growth	*Cymbopogon winterianus*	Should be excluded from Fusarium wilt control strategies
		*Salvia hispanica*	
		*Satureja horvatii*	
Phytotoxicity at high doses	Effective only at concentrations causing plant damage or growth suppression	*Syzygium aromaticum* (≥10%),	Highlights the need for dose optimization and encapsulation
		*Origanum vulgare* (high doses),	
		*Capsicum annuum*	

**Table 3:** Volatile compounds, evaluated against FOL in tomato.

Compound	Chemical Group	Antifungal Activity of the Main Compound	Reference(s)
Cinnamyl acetate	Phenylpropanoid ester	Moderate mycelial growth inhibition in vitro; consistently weaker than cinnamaldehyde; no in vivo or greenhouse evaluation reported	[30]
Cinnamaldehyde	Phenylpropanoid	Strongest cinnamon-derived compound with high inhibition at low doses in vitro; nanoemulsion formulation contributed to ~50–60% reduction in fruit disease severity under in vivo conditions	[30]
Camphene	Monoterpene hydrocarbon	MIC vs. *F. oxysporum* = 0.16 ± 0.03 mg/mL (in vitro)	[25]
Camphor	Oxygenated monoterpene	MIC vs. *F. oxysporum* = 0.11 ± 0.02 mg/mL (in vitro)	[25]
Carvacrol	Monoterpenoid phenol	Strong mycelial inhibition (EC_100_ ≈ 166 µg·mL^−1^); fungistatic at lower doses. Seed treatment (1200 µg·mL^−1^) reduced AUDPC by ~54% and wilt incidence; safe for germination	[27]
Carvone	Oxygenated monoterpene	Up to 90.98% mycelial growth inhibition at 500 ppm (partition plate assay)	[39]
Citral (neral + geranial)	Monoterpenoid aldehydes	Highly fungicidal. In vitro: MIC/MFC ≈ 512 µg·mL^−1^; complete inhibition at ≥500 µg·mL^−1^ and ≥1.5% (*v*/*v*); ≥84% radial inhibition at 0.4–2 mL·mL^−1^. In vivo/greenhouse: soil drench reduced wilt severity by 46–54% (control 61.7% → 28.3–33.3%); strong induction of *PR* genes (chitinase, β-1,3-glucanase, TLP). Nano-citral (chitosan NPs) showed lower efficacy than free citral	[33,61,62,63]
Citronellal	Monoterpenoid aldehyde	Moderate inhibition (~40–50%); fungistatic behavior in vitro (~46% inhibition at 0.5 µL·mL^−1^); no in vivo validation	[56]
Citronellol	Monoterpenoid alcohol	Stronger fungicidal activity than citronellal; low IC_50_ (0.207 µL·mL^−1^); 100% inhibition at ≥0.5 µL·mL^−1^ and at 500 ppm (in vitro); no greenhouse data reported	[39,56]
Linalool	Monoterpenoid alcohol	Antifungal activity reported in vitro, but no quantitative MIC/MFC values against FOL, and no in vivo or greenhouse validation	[64]
Eugenol	Phenylpropanoid	Moderate inhibition; largely fungistatic in vitro (EC_50_ = 187.5 µg·mL^−1^; EC_100_ = 374.9 µg·mL^−1^); strong inhibition of mycelial growth and conidial germination; highest combined in vitro and greenhouse efficacy among tested phenylpropanoids	[27,42]
Geraniol	Monoterpenoid alcohol	Very strong antifungal activity; fungicidal at low concentrations. In vitro: dose-dependent inhibition (up to ~68% at 2 mL·mL^−1^); IC_50_ = 0.144 µL·mL^−1^, IC_90_ = 0.610 µL·mL^−1^, 100% inhibition ≥0.5 µL·mL^−1^. In vivo: soil drench suppressed Fusarium wilt comparable to a chemical fungicide	[39,56,62]
Nerol	Monoterpenoid alcohol	Complete inhibition (100%) of *F. oxysporum* growth at ≥1.5% (*v*/*v*)	[33]
Thymol	Monoterpenoid phenol	Complete inhibition of conidial germination at all tested concentrations; MIC_50_ ≈ 295 µg·mL^−1^	[42]
trans-Anethole	Phenylpropanoid ether	IC_50_ = 0.14 mg·mL^−1^ (direct contact assay)	[51]
α-Pinene	Monoterpene hydrocarbon	No inhibition of mycelial growth; partial inhibition of conidial germination only	[42]
β-Caryophyllene	Sesquiterpene	MIC vs. *F. oxysporum* = 0.13 ± 0.01 mg·mL^−1^ (in vitro)	[25]

**Table 4:** Nanoformulations of EOs/components against FOL in tomato.

Active EO/Compound (Merged)	Nano-Carrier and Formulation	Antifungal Efficacy	The Main Advantage of Using Nanoformulation	Reference(s)
Citral―nano vs. free (comparator)	Citral–chitosan nanoparticles (CCNPs); ionic gelation; low encapsulation efficiency + free citral comparator	Nano-citral showed weak in vitro antifungal activity with no reported plant-level validation, whereas free citral exhibited strong fungicidal activity in vitro and consistent disease suppression in vivo	Nanoformulation improved handling and controlled release, but reduced biological efficacy due to low loading and slow release	[61]
Cinnamon EO (cinnamaldehyde-rich) + *Annona squamosa* seed extract	Botanical oil-in-water nanoemulsion	Strong in vitro inhibition and moderate to high disease control under in vivo conditions	Improved dispersion, stability, and bioavailability compared with bulk botanical oils	[30]
Volatilomes (phyto-fumigant system): *Mentha spicata* vs. *Cymbopogon citratus*	Vermiculite-immobilized volatilomes	Volatilomes caused high in vitro growth inhibition; *M. spicata* additionally achieved very strong disease suppression in vivo, while *C. citratus* showed lower efficacy	Sustained vapor release and effective diffusion in soil and enclosed environments	[31]
Clove EO (*Syzygium aromaticum*; eugenol-rich)―multiple carriers	Oil-in-water nanoemulsion; β-cyclodextrin inclusion complex; β-cyclodextrin microcapsules	Nano-formulated clove EO showed markedly enhanced in vitro antifungal activity compared with free EO; no plant-level validation was reported	Improved dispersibility, stability, and sustained release, leading to stronger antifungal effects	[32,46,47]
Clove EO + Lemongrass EO (1:1)	Non-ionic nanoemulsion	Nanoemulsion demonstrated higher in vitro potency and substantial disease suppression in vivo, without observable phytotoxicity	Reduced effective dose, faster fungicidal action, and improved soil performance	[65]
Mexican oregano EO (*Lippia berlandieri*)	β-cyclodextrin microcapsules	Microencapsulation resulted in stronger and more persistent in vitro antifungal activity compared with free EO; no in vivo data available	Improved persistence and controlled release	[46]

## Data Availability

The original contributions presented in this study are included in the article. Further inquiries can be directed to the corresponding author.

## Abbreviations

The following abbreviations are used in this manuscript:

AgNPs	Silver nanoparticles
AUDPC	Area under the disease progress curve
β-CD	β-Cyclodextrin
CWDEs	Cell wall–degrading enzyme
EC_50_	Effective concentration causing 50% inhibition
EC_90_	Effective concentration causing 90% inhibition
EO	Essential oil
FOL	*Fusarium oxysporum* f. sp. *Lycopersici*
GC-MS	Gas chromatography–mass spectrometry
IC_50_	Inhibitory concentration causing 50% inhibition
ITS	Internal transcribed spacer
LOX	Lipoxygenase
MTG	mean time to germination
MIC	Minimum inhibitory concentration
MFC	Minimum fungicidal concentration
NPs	Nanoparticles
PDA	Potato dextrose agar
PR genes	Pathogenesis-related genes
PEDC	Percent efficacy of disease control
PDS	Percent disease severity
PDI	Percent disease index
PRISMA	Preferred Reporting Items for Systematic Reviews and Meta-Analyses
ROS	Reactive oxygen species
TLP	Thaumatin-like protein
VOCs	Volatile organic compounds
ZnO	Zinc oxide
